# Task-Modulated Oscillation Differences in Auditory and Spoken Chinese-English Bilingual Processing: An Electroencephalography Study

**DOI:** 10.3389/fpsyg.2022.823700

**Published:** 2022-05-30

**Authors:** Yuxuan Zheng, Ian Kirk, Tengfei Chen, Minako O’Hagan, Karen E. Waldie

**Affiliations:** ^1^School of Psychology, The University of Auckland, Auckland, New Zealand; ^2^Centre for Brain Research, The University of Auckland, Auckland, New Zealand; ^3^School of Physical and Mathematical Sciences, Nanjing Tech University, Nanjing, China; ^4^School of Cultures Languages and Linguistics, The University of Auckland, Auckland, New Zealand

**Keywords:** overt interpreting, EEG oscillations, theta-gamma coupling, time-frequency power, bilingualism

## Abstract

Neurophysiological research on the bilingual activity of interpretation or interpreting has been very fruitful in understanding the bilingual brain and has gained increasing popularity recently. Issues like word interpreting and the directionality of interpreting have been attended to by many researchers, mainly with localizing techniques. Brain structures such as the dorsolateral prefrontal cortex have been repeatedly identified during interpreting. However, little is known about the oscillation and synchronization features of interpreting, especially sentence-level overt interpreting. In this study we implemented a Chinese-English sentence-level overt interpreting experiment with electroencephalography on 43 Chinese-English bilinguals and compared the oscillation and synchronization features of interpreting with those of listening, speaking and shadowing. We found significant time-frequency power differences in the delta-theta (1–7 Hz) and gamma band (above 30 Hz) between motor and silent tasks. Further theta-gamma coupling analysis revealed different synchronization networks in between speaking, shadowing and interpreting, indicating an idea-formulation dependent mechanism. Moreover, interpreting incurred robust right frontotemporal gamma coactivation network compared with speaking and shadowing, which we think may reflect the language conversion process inherent in interpreting.

## Introduction

Over half of the world’s population can speak two languages or more (Grosjean, 1994), and the practice of alternatively using two languages is referred to as bilingualism (Weinreich, 1953). It is believed that the brain’s ability to switch between two languages may also generalize to non-linguistic tasks requiring selective attention and inhibition (cf. Lehtonen et al., 2018). Among the many bilingual switching activities, interpreting is perhaps the most interesting. Interpreting refers to a process, in which a first and final rendition in another language is produced on the basis of a one-time presentation of an utterance in a source language (Pöchhacker, 2004, p. 11). Interpreting is one of the most cognitively demanding language tasks for the human brain, involving decoding the source language, storing the information in working memory, reformulating the information and articulating in the target language, all having to be completed within a tight time window measurable in seconds (Christoffels et al., 2006; Elmer et al., 2010; Klein et al., 2018).

Although it has been suggested that investigating the neural mechanisms of interpreting would benefit the area of neurolinguistics as a whole (García, 2013), these remain scarcely investigated. Of the existing neurolinguistic research on interpreting, the majority are localization studies using functional magnetic resonance imaging (Lehtonen et al., 2005; Elmer, 2016; Elmer et al., 2014; Hervais-Adelman et al., 2015a,b, 2017; Zheng et al., 2020), positron emission tomography (Klein et al., 1995; Price et al., 1999; Rinne et al., 2000; Tommola et al., 2000), functional near-infrared spectroscopy (Quaresima et al., 2002; Lin et al., 2018a,b; Ren et al., 2019), and diffusion tensor imaging (Van de Putte et al., 2018). Though findings vary, brain structures that have been consistently identified as being involved in interpreting include the dorsolateral prefrontal cortex, pars triangularis and supramarginal gyrus.

As such, although the cerebral “where” question of interpreting has been partly answered, the answers to the “how” question of interpreting remain elusive. For example, we still do not know why interpreting is difficult, what the functional features underlying interpreting are, and whether they are intrinsically different from those of other bilingual activities. To further demystify the neural workings of interpreting, some pioneering researchers conducted experiments with electroencephalography (EEG), which has the benefit of helping us understand the timing of neural events during language processing. Early EEG studies of interpreting mainly showed a temporal beta-band oscillation associated with word interpreting, larger centroparietal theta increase and frontal alpha decrease for interpreting low-frequency words relative to high-frequency words, and wider brain activations for first language (L1) to second language (L2) interpreting than for L2–L1 interpreting (Petsche et al., 1993; Kurz, 1995; Grabner et al., 2007). Note, however, that these early studies all adopted the “mental interpreting” or the “typing response” design paradigm (i.e., silently interpreting the words or typing down the answer while seeing the stimulus words, both without any form of overt speaking), which are not in line with real-life interpreting activities. This makes the validity of the early studies and their results problematic.

Revolutions of experiment design began soon after researchers realized the flaws in ecological validity in the early studies. Janyan et al. (2009) made the first attempt to test overt spoken interpreting with EEG. They asked 22 Bulgarian-English bilinguals to interpret visually presented L2 words into L1, taking cognates/non-cognates and word concreteness as the independent variables. The results showed that interpreting cognates elicited centrotemporal N400 component and that the word concreteness effect was only associated with cognates. Later, Christoffels et al. (2013) adapted this paradigm and measured the event-related-potentials (ERPs) of 57 Dutch-English bilinguals when they were doing two-way overt word interpreting and naming. Their experiment revealed that participants began to differentiate the direction of interpreting at around 200 ms, and activation reflecting the meaning of words occurred at 300 ms. They also found that L1–L2 interpreting elicited more P2 component while L2–L1 interpreting brought about more N400 component. Following this, Jost et al. (2018) examined the ERP of 15 French-English bilinguals in such overt tasks as two-way word interpreting, L1 word generation and L2 word generation. The results showed that the differences between word generation and word interpreting were manifested in the 424–630 ms time window. They also noted that backward interpreting (BI; L2 to L1) was easier than forward interpreting (L1 to L2), as the latter incurred wider brain activations. But this finding was not completely unchallenged. Dottori et al. (2020) compared the neurophysiological signatures of word interpreting between professional Spanish-English simultaneous interpreters and bilingual Spanish-English non-interpreter controls. Their results showed that it was the BI (L2–L1) that triggered the more widespread neural activation among professional interpreters. They also found a consistently higher delta-theta power of professional interpreters than by the controls. Pérez et al. (2022) implemented a similar EEG study on Spanish-English bilinguals and compared the oscillation differences in two-way word translation tasks. Their results mainly showed that, compared with backward translation, forward translation yielded higher frontal theta in an early window, lower central beta in a later window, and a positive early theta-behavioral data association. In short, testing overt spoken interpreting has been proved not only feasible but also fruitful. The N400 component was identified as being closely related to interpreting, and more evidence showed that BI (L2–L1) is easier than forward interpreting (L1–L2) than the reverse way. In contrast to these ERP studies, to our knowledge there is no oscillation research on overt spoken interpreting.

Despite the progress made in the experiment paradigm, there are two other gaps in the existing literature on interpreting research: (1) there are very few sentence-level studies; and (2) most of the sentence-level studies were on the localization side (e.g., Lehtonen et al., 2005; Hervais-Adelman et al., 2015a,b; Zheng et al., 2020). Thus, there is a need for oscillation research on sentence-level overt interpreting.

Another important problem with previous research lies in the experiment mode. Nearly all prior studies chose to present their stimuli visually to the participants, which, strictly speaking, should be called “sight translation” in reference to the immediate oral rendition of the written text, as a specific mode of interpreting (He et al., 2017). As word interpreting and sentence interpreting do not share the same neural mechanisms (Klein et al., 1995; García, 2013; Muñoz et al., 2019), and that the human brain processes visual and acoustic information differently (Baddeley, 2007), it is an important next step to examine the EEG oscillation features of sentence-level interpreting in an auditory-spoken design.

Against this backdrop, we conducted an EEG study that aims to reveal the functional workings of sentence interpreting with an auditory-spoken design. Specifically, we implemented four language tasks in the EEG environment: (i) L2 listening (L2L); (ii) L1 speaking (L1S); (iii) L2 shadowing (L2SH); and (iv) BI. Our aim was to determine the dominant EEG frequency bands while participants are interpreting. We also addressed whether the frequency bands were unique to interpreting or general to other language tasks. Though our study was exploratory regarding hypotheses, particularly as to the language conversion process specific to interpreting, some related studies are noteworthy. For example, Grabner et al. (2007), in a silent experiment design, found a theta increase for BI; Dottori et al. (2020) partly corroborated this and further reported a delta-theta (1–8 Hz) increase in an overt interpreting task, indicating that the theta band (4–7 Hz) or even the broader delta-theta band (1–8 Hz) is closely related to the word interpreting process. On the other hand, as noted, sentence interpreting involves much more semantic and syntactic integration (García, 2013). We therefore predict that, apart from theta band activation, overt sentence-level BI may also incur stronger syntactic-related alpha/beta band (Davidson and Indefrey, 2007; Bastiaansen et al., 2010; Segaert et al., 2018) activation, as well as semantic-related gamma band (Hald et al., 2006; Wang et al., 2012; Rommers et al., 2013; Bastiaansen and Hagoort, 2015) activation than other language tasks.

## Materials and Methods

### Participants

Forty-six subjects were recruited for the current study. One participant did not finish the experiment and another two were excluded due to noisy data. The remaining forty-three subjects were used for the current data analysis.

Participants were bilingual Chinese (L1) – English (L2) non-interpreters with varying degrees of L2 proficiency, roughly gender-balanced (female = 21, male = 22), aged between 19 and 36 years (*M* = 26.05, SD = 4.55). They began learning English as second language from a mean age of 8.12 years, SD = 3.16, and have been learning English for 17.6 years on average, SD = 4.59. Their average stay in English-speaking countries was 3.43 years (SD = 3.79). None had a history of brain injury, and all had normal hearing and speaking abilities according to their self-reported answers in the questionnaire.

All participants gave written, informed consent and were compensated with a 20-dollar grocery voucher for their participation. Experiments were implemented in accordance with the Declaration of Helsinki. The study was approved by The University of Auckland Human Participants Ethics Committee (Ref. 022991).

### Tasks and General Procedures

Before the EEG session, participants were given time to read through the Participant’s Information Sheet and the Consent Form. Then they were asked to complete the Oxford Placement Test (within 30 min) and a demographic questionnaire that mainly covered their language backgrounds.

**FIGURE 1:**
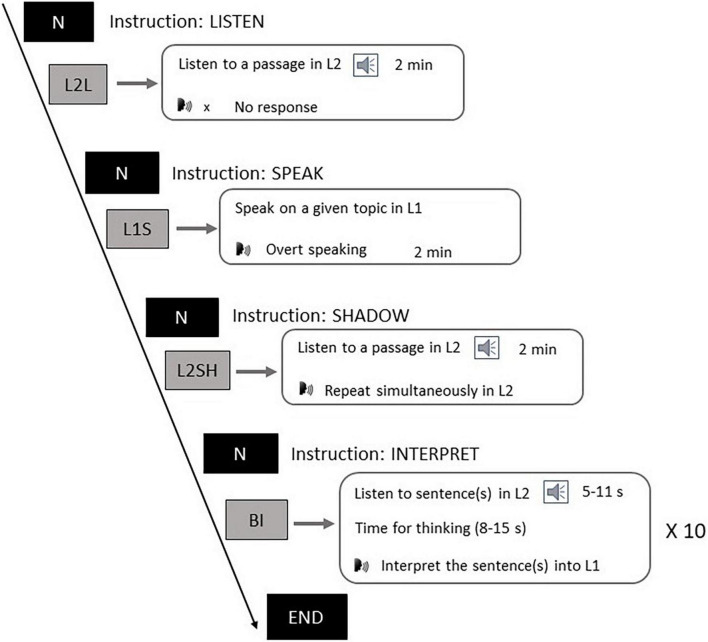
Experiment tasks.

The experiment consisted of four language tasks, as shown in Figure 1. Participants first listened to the general instructions of the whole experiment which detailed what to do in each task. Next, participants listened to the instructions on the L2L task and subsequently listened to the auditory stimuli. During this task participants were required to comprehend the news without needing to give other responses. The L2L task was designed to test the brain oscillations in a sensory task. In the L1S task participants first heard instructions on a topic they were going to talk about. After that they had 1 min to prepare. A beep sound popped up at the end of the preparation, reminding them to start speaking until the second beep came up to stop them. This task was aimed at examining the neural oscillation features of L1 motor process. In the L2SH task participants listened to instructions first, and then they were required to listen to a paragraph in English and concurrently repeat every word of it. The goal of the L2SH task was to probe the oscillations underlying L2 motor process. Each of the first three tasks consisted of a long single trial of 2 min. In the BI task participants were asked to listen to (a) short English sentence(s), after which they had several seconds for comprehension, and then they had to interpret the sentence(s) into Chinese upon hearing the first beep and stop upon the second. The BI procedure repeated ten times for ten different sentence groups and was designed to test if there was a language conversion mechanism manifested in the oscillations. Such experiment design was adapted from that used in Hervais-Adelman et al. (2015b), and here we added a speaking task to include more possibilities of comparison. The whole experiment was conducted in an EEG setting using E-Prime 2.0 (Psychology Tools, Inc., Pittsburgh, PA, United States) and participants’ verbal outputs were recorded with a voice recorder.

The EEG recordings were conducted in an electrically shielded room (IAC Noise Lock Acoustic – Model 1375, Hampshire, United Kingdom) using 128-channel Ag/AgCl electrode nets (Tucker, 1993) from Electrical Geodesics Inc (Eugene, OR, United States). EEG was recorded continuously (1,000 Hz sample rate: 0.1–400 Hz analog bandpass) with Electrical Geodesics Inc. amplifiers (300-MΩ input impedance). Electrode impedances were kept below 40 kΩ, an acceptable level for this system (Ferree et al., 2001). Common vertex (Cz) was used as a reference. During the EEG, participants were comfortably seated in a chair 60 cm away from the screen. As there was no visual information involved, we asked the participants to close their eyes during the experiment, both to reduce noise in data from eye movements and to eliminate visual distractions (even if fixation has been widely used in previous studies to control eye movement, there is still a + sign in the middle of the screen that would potentially activate the brain’s visual system).

To familiarize participants with these tasks a practice session was administered before the real experiment. To avoid fatigue-induced performance deteriorations, the four tasks were designed to appear in a balanced sequence so that each task was used as the first condition for an equal number of times. Between the second and third task there was a break time (about 10 min). The whole experiment lasted for about 60 min.

### Materials

A written version of the Oxford Placement Test^1^ was used for the testing of participants’ English proficiency before the EEG experiment. The experiment consisted of three custom-made English audio clips. The first clip was a passage of news, lasting for 2 min and was used for the L2L condition (162 words per minute). The second clip was also a 2-min news excerpt but was much slower (95 words per minute) and was used for the L2SH condition. The third clip was made up of ten groups of English sentences with varying length (11 to 33 words, mean = 20.5, SD = 8.18), played at a mean speed of 153 words per minute, and used for the BI condition. All sentences were in the simple declarative form (e.g., S1 for interpreting: *The United Kingdom research councils are establishing their first overseas office in Beijing*.) so that syntactic complexity as a confound variable can be ruled out. The full list of sentences used for BI can be found in section “Appendix.”

### Data Analysis

#### Preprocessing

EEGlab v2020.0 (Delorme and Makeig, 2004) was used for the preprocessing of the raw data. The raw data was first downsampled to 250 Hz and high-pass filtered at 1 Hz. Then the data was cleaned using EEGlab plugins Cleanline (v1.04) and Clean_rawdata (v.2.2). Next, bad channels were interpolated and data re-referenced to the average of all channels. After this, Independent Component Analysis (ICA) was performed based on the EEGlab default algorithm of “runica,” and the number of principal components (PCs) to decompose was set to be equal to the number of channels. Finally MARA (Winkler et al., 2011) was applied to remove artifactual components using default parameters. Considering the potential motor artifacts which the overt tasks may produce, we doublechecked the remaining component map and removed those that looked suspicious. After this we again inspected the time domain signal and removed any portion of data that looked noisy. Specific artifact removal and noisy data exclusion criteria are as follows. Any signal portion with a 5-s flatline duration or longer was removed, and a channel with less than 85% self-reconstruction based on other channels was considered abnormal and then removed. A channel was interpolated if the line noise was four standard deviations higher than its signal based on the whole channel population. Signal bursts whose variance was 10 standard deviations higher than the calibration data were considered missing and were removed. A maximum of 25% of contaminated channels in a certain time window for data repairment was considered tolerable, otherwise that window was removed. On average, bad channel interpolation rate was 5.84%, 57 independent components were computed, and 4% of the original signal (about 52 s) was removed, across subjects.

Then we extracted epochs of 60 s (0–60 s) from the preprocessed data for the L2L, L1S, and L2SH conditions, respectively. Since the BI task was in the form of ten consecutive sentences, we concatenated the preprocessed sentence data and then extracted a 60-s epoch in line with the other three data segments. The epoch extraction of BI sentences was synchronized with the overt interpreting. Data exclusion was based on the type of blanks in the data segment, such that blank periods where the brain was not involved in language conversion (e.g., after the interpreting finishes but before the stopping beep sounds) were removed, while those during which language conversion happens (e.g., pauses in the middle of interpreting) were kept. We did not take individual participant’s interpreting speed into account, because when we classified the types of blanks speed was no more concern for data exclusion. A 2-s baseline was drawn from the resting state data for each individual, respectively.

#### Time-Frequency Analysis

The functional mechanism of the four bilingual processing tasks in which we were most interested was indexed by time-frequency power values. We analyzed the time frequency power dynamics of all datasets using the method of complex Morlet wavelet convolution in Matlab R2019b (The MathWorks, Inc., Natick, MA, United States). The 2-s resting state dataset drawn from each individual subjet’s preprocessed data was used as the baseline window for that particular subject accordingly. Then the convolution frequencies were created as a linearly spaced vector ranging from 1 to 40 Hz in 30 steps. The key parameter of the complext Morlet wavelet, i.e., the full width at half maximum, was defined as a vector from 300 ms to 600 ms logarithmically spaced in 30 steps, in order to achieve an ideal tradeoff between time and frequency precision. The task-induced time-frequency data was then decibel normalized by the baseline as a way to reveal the task-relevant dynamics. We then averaged all the data across 43 participants in one condition to obtain group results for that particular task. The raw time-frequency power data was downsampled at a time interval of 200 ms, which had no impact on the time-frequency resolution but saved much storage space of the computer.

We then performed condition-wise subtractions on the group-level time-frequency data in order to see if there were frequencies unique to a specific task. Such subtraction analysis has been adopted effectively by earlier research (Klein et al., 1995; Rinne et al., 2000; Perani et al., 2003; Wang et al., 2018; Muñoz et al., 2019). As such, we adopted the non-parametric cluster-based permutation test (Maris and Oostenveld, 2007) for statistical testing. First, the power values of the frequencies of interest (1–40 Hz) in each channel and time point within the 0–60 s window were clustered depending on if they exceed the dependent *t*-test threshold. This was repeated 1,000 times through random partitions in which the labels of the two conditions were shuffled. Then the largest cluster size was counted for all 1,000 partitions. After that the distribution of the 1,000 largest cluster sizes was calculated and a 95 percentile identified as the true threshold (i.e., the Monte Carlo *p*-value) to reject the null hypothesis. Lastly the permuted threshold was applied to the original data, and clusters with size values higher than such threshold were kept while those lower than the threshold were deemed chance results, thus non-significant.

Note that the time-frequency analysis was implemented in a lobe-wise fashion, i.e., for a certain lobe the data was averaged across all electrodes in that lobe to obtain a grand result. For instance, the time-frequency result for the frontal lobe was obtained by averaging the individual results across channel F1 to F10. The condition-wise subtractions were conducted within the same lobe, while the more dynamic cross-region oscillation analysis was attended to in the later section for cross-frequency-coupling.

### Behavioral Analyses and Preliminary Findings

Demographical and behavioral data were analyzed using IBM SPSS version 26 (SPSS Inc, Chicago, IL, United States). We measured participants’ L2 proficiency and transcribed and marked their oral interpreting output as the main behavioral index. Interpreting performance was measured as the ratio of correctly interpreted words to the total words in the original sentence. For example, if a sentence contained 12 content words (i.e., excluding articles such as *the*) and the participant correctly interpreted four of them, then his/her interpreting accuracy was 33.33%. The average score of the 43 participants in the Oxford Quick English Placement test was 44.37 (full marks = 60), SD = 7.32. The mean accuracy of their interpreting performance was 54.59%, SD = 0.15.

Correlations were then performed on the behavioral index and participants’ language background. There was a significant correlation between participants’ L2 proficiency and their interpreting accuracy, *r*(41) = 0.67, *p* < 0.01. There was also a significant correlation between the time length that participants spent living in English-speaking countries and their interpreting performance, *r*(41) = 0.46, *p* < 0.01. There were no significant correlations between interpreting performance and age, age of L2 acquisition and L2 learning years, all *p*s > 0.05.

## Results

### Time Frequency Results

**FIGURE 2:**
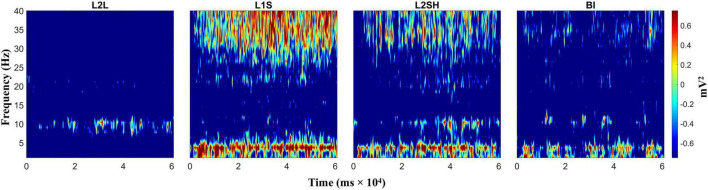
Raw time-frequency power in all conditions (language tasks). From left to right: L2 listening (L2L), L1 speaking (L1S), L2 shadowing (L2SH), and backward interpreting (BI), respectively. TF power values are gained by task/baseline division.

We first evaluated the raw time-frequency dynamics in each condition, with the intention to identify the dominant frequency bands in the four language tasks. We did not have *a priori* hypotheses on any regions of interest, so we did this for the frontal, occipital, parietal, and temporal regions, respectively, by averaging the TF dynamics across all electrodes in that specific region. The results are not qualitatively different for the four lobes, and one example is shown in Figure 2.

Figure 2 shows a marked TF power difference between L2L and the other three conditions, meanwhile the last three language tasks also demonstrated slight TF variations between each other, though the general activation pattern was similar. Specifically, L2L was dominated by alpha activation (around 10 Hz), while L1S, L2SH, and BI mainly elicited coactivation between delta-theta and gamma bands. L1S had the highest power values, most sustained activation and widest frequency ranges. L2SH in contrast, showed less activation in terms of frequency coverage and consistency. BI was even less in all the activation indexes. Note, however, that both L2SH and BI elicited slightly stronger alpha activation than L1S. Particularly in BI, the theta and alpha activations seemed to appear roughly in an alternating order.

**FIGURE 3:**
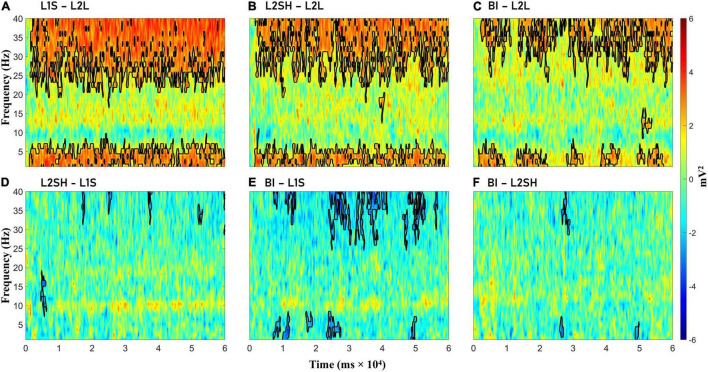
Condition-wise time-frequency power differences. **(A)** L1 speaking (L1S) minus L2 listening (L2L). **(B)** L2 shadowing (L2SH) minus L2L. **(C)** Backward interpreting (BI) minus L2L. **(D)** L2SH minus L1S. **(E)** BI minus L1S. **(F)** BI minus L2SH. Statistically significant differences are marked with black lines.

Figure 3 illustrates the condition-wise TF power subtractions. We observed significant gamma and delta-theta increase in the three overt tasks (i.e., L1S, L2SH, and BI) compared to L2L, but virtually no significant differences among the three tasks themselves, except in BI minus L1S, where the former showed some inconsistent but significant gamma and delta-theta power decrease than the latter. L2SH and BI also had consistent higher alpha power than L1S, but the differences were non-significant.

As L1S, L2SH, and BI all involved overt speaking, we do not know if the observed results in Figure 3 were real effects or muscle artifacts. Therefore we did a further PCs analysis to extract the main topography for the three overt tasks. If the three tasks share the same PCs, then the TF results are nothing but muscle artifacts; if they involve distinct PCs then it is very likely that the results are real effects. For comparison we also analyzed the PCs in L2L.

### Principal Components Analysis

**FIGURE 4:**
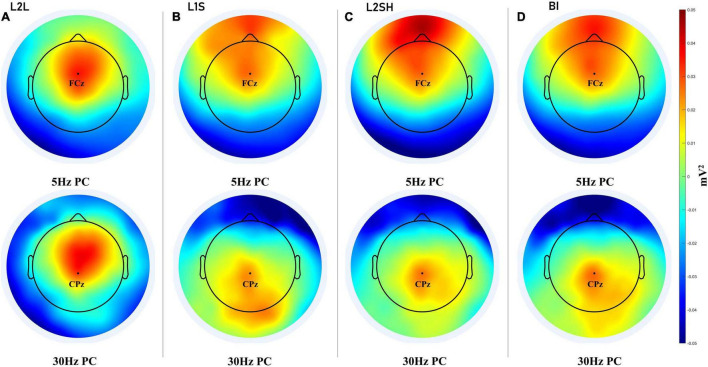
PCA results for all language tasks. **(A)** PC for 5 and 30 Hz in L2L. **(B)** PC for 5 and 30 Hz in L1S. **(C)** PC for 5 and 30 Hz in L2SH. **(D)** PC for 5 and 30 Hz in BI. For each panel channel FCz and channel CPz are marked for the convenience of comparison.

We first narrowband filtered the data at 5 Hz and 30 Hz, respectively, with a full width at half maximum of 2 Hz, so that the filtered data fell into the delta-theta band (3–7 Hz) and the lower gamma band (28–32 Hz). Then we extracted the PCs of the filtered data using the method of eigendecomposition. Finally we plotted the first PCs in the these tasks which are presented in Figure 4.

The principal component analysis (PCA) results first showed a clear-cut distinction between L2L and the other three tasks. L2L elicited the coactivation of frontal theta and frontal gamma, while the other three conditions either invovled a frontal theta and a parietal gamma or a prefrontal theta and a parieto-occipital gamma. Specifically, L1S was characterized by the most extensive parietal and bilateral occipital gamma coupled by a frontal theta, while L2SH was marked by a strong prefrontal theta and a fairly limited centroparietal gamma. BI had more right-lateralized parietal and occipital and even temporal gamma, and a frontal theta that is not very strong. This result further distinguished the three overt tasks, but to unveil the mechanisms of each task more analysis is needed.

### Cross-Frequency Coupling and Synchronization

**FIGURE 5:**
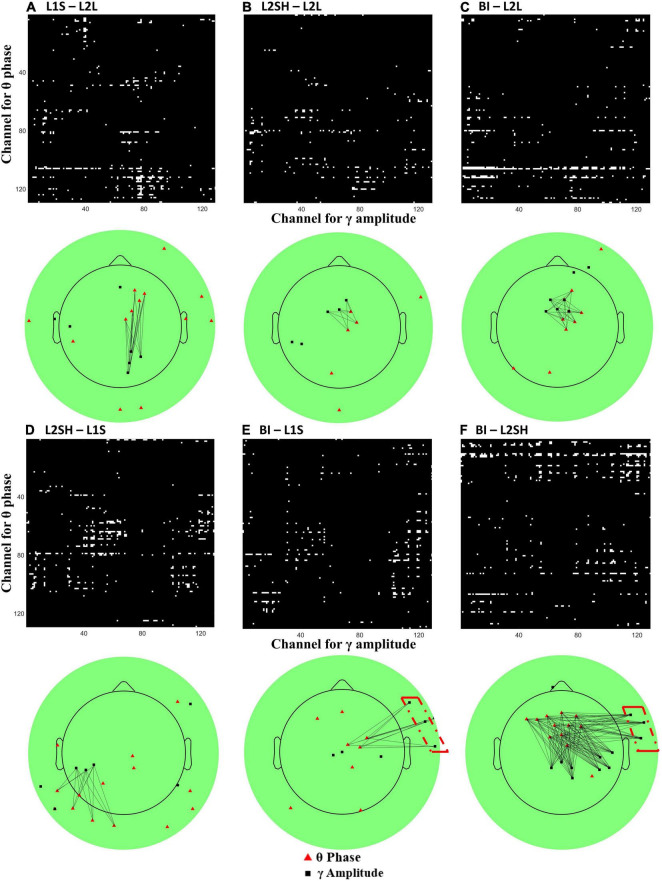
All-to-all theta-gamma coupling (TGC) synchronization map and its topography. **(A)** L1 speaking minus L2 listening. **(B)** L2 shadowing minus L2 listening. **(C)** Backward interpreting minus L2 listening. **(D)** L2 shadowing minus L1 speaking. **(E)** Backward interpreting minus L1 speaking. **(F)** Backward interpreting minus L2 shadowing. On each panel the upper half is channel-to-channel theta (*Y* axis) and gamma (*X* axis) coupling map, where white pixels represent TGC values surviving a threshold of two standard deviations above the median, and the lower half is the projection of the upper map onto the 128-channel EEG scalp, where the red triangles sit in the position of electrodes for theta activation and the black squares gamma activation. The black lines mark the potential synchronizations between electrodes. Note that only clusters (neighboring electrode numbers > 3) are linked in the plot for illustration purposes. The red dash-dot boxes on **(E,F)** mark the unique synchronization of BI compared to L1S and L2SH.

Since the time-frequency results did not differentiate BI from other overt spoken tasks, we sought to find BI-specific features in other ways. It has been proposed that, aside from the static individual frequency bands’ activities, the interactions between different frequency bands, often known as cross-frequency coupling (CFC) or phase-amplitude coupling, may even better reflect the functional dynamics of the brain (Canolty and Knight, 2010). Given that theta and gamma bands have been identified as the frequencies of interest in the aforementioned time-frequency results, we further performed CFC analysis on these two bands across all electrodes, with the intention to find more nuanced synchronization mechanisms for each condition. We first selected one channel (beginning from number 1) and narrowband filtered its data at theta band (3–7 Hz) and gamma band (30–60 Hz). Then we extracted the theta band phase angles and the gamma band amplitude values using Hilbert transform, eulerizing the two numbers to get a CFC value. After that we did a permutation test (number of iterations = 2,000) on the CFC value to eliminate chance results and assigned the permuted value to that particular channel pair. We moved on to the next channel and repeated this analysis until we finished the loop over all 128 electrodes. Following that we averaged the data across all participants and obtained a group-level synchronization map for each condition. Lastly we applied a stringent threshold (2 standard deviations above the median) to the map in order to reveal the robust hubs of synchronization. The results are presented in Figure 5.

Figure 5 shows two salient condition-wise differences regarding theta-gamma coupling (TGC) features. First, the three plots in the first row show a consistent right hemisphere central or frontocentral theta activation. Figures 5B,C show even similar TGC directions compared with Figure 5A. The second feature is that in the second row the TGC synchronization exhibits more disparities than in the first row. Specifically, L2SH elicited more extensive TGC synchronization than L1S (Figure 5D), while BI incurred more clustered TGC than L2SH (Figure 5F). Compared with L1S and L2SH, BI consistently triggered extra right temporal gamma synchronization (highlighted with red dash-dot boxes in Figures 5E,F).

## Discussion

To explore if there is a unique oscillation mechanism for the interpreting activity, we conducted an EEG experiment on 43 Chinese-English bilingual non-interpreters and analyzed their oscillation dynamics in 4 tasks: listening to an excerpt of English news (L2L); speaking on a given topic in Chinese (L1S); listening to an excerpt of English news and concurrently repeating it (L2SH); and interpreting English sentences into Chinese (BI). To our knowledge, the current study is the first EEG experiment on auditory-oral sentence-level interpreting. Such design is novel perhaps due to the sensitive nature of the EEG technique to motor artifacts, i.e., head and muscle movement caused by speaking can affect the EEG signal. We tried to eliminate the artifacts by removing the muscle components both with ICA and by handpicking the noisy parts of the data after preprocessing. The analysis of the data presented novel findings on both the behavioral side and the oscillatory side.

The behavioral results revealed that participants’ interpreting performance was significantly correlated with their L2 proficiency and L2 exposure, but not with their age, age of L2 acquisition and L2 learning years. Our results on the one hand corroborated previous findings on the positive relation between L2 proficiency and interpreting/translation performance (De Groot and Poot, 1997; Tzou et al., 2012; Mayor, 2015; Chen et al., 2020), on the other hand provided novel evidence on the association between interpreting performance and age, age of L2 acquisition and L2 learning years, which have never been reported before. Since interpreting performance was not influenced by age-related factors, we therefore regard interpreting as an activity that relies more on intensive training than on biological aptitude.

With respect to oscillations, we expected to see the delta-theta band activation in the BI condition (as has been found in previous word level research), stronger alpha/beta band activation for syntactic binding and gamma band activation for semantic integration in BI relative to other three language tasks. The results revealed different patterns in TF power and CFC.

First of all, condition-wise TF subtractions revealed significant differences between three overt tasks (L1S, L2SH, and BI) and L2L in the gamma band and the delta-theta band. On the one hand, these results confirmed part of our hypothesis, including the delta-theta band and the gamma band activations for BI. On the other hand, however, such TF patterns were not unique to BI, instead they looked quite similar across the three overt tasks. Hence we conducted a PCA on the theta and gamma band data, respectively, in trying to extract the topography of the three overt tasks. The PCA results revealed a frontal delta-theta and a parietal gamma in the three tasks, with varying size and energy that were distinguishable for each task. Following this, we extracted all-to-all TGC synchronization difference maps as an attempt to locate robust hubs for task-related synchronization. The results showed a consistent central/frontocentral theta synchronization for three overt tasks (L1S, L2SH, and BI) as compared to L2L. We therefore speculate that the frontocentral theta coactivation is an indispensable part of overt speaking-related tasks. Moreover, both L2SH and BI incurred similar synchronization networks, i.e., between right frontocentral theta and mid-frontal gamma, which was different from the pattern in L1S. Considering that in both L2SH and BI participants were fed with well-formed ideas from others, while L1S involved the stage of “message generation” on ones’ own (Levelt, 1989), it is reasonable to infer that genuine idea forming is manifested in synchronization between right frontocentral theta and right parieto-occipital gamma, whereas reformulating other’s ideas lies in the network of right frontocentral theta and midfrontal gamma coupling.

Another important finding of the current study is that in between the three overt tasks, BI consistently triggered more right frontotemporal gamma synchronization than the other two. This is in general agreement with the PCA result for gamma in BI, which was more right lateralized than L1S and L2SH. Therefore the right frontotemporal gamma coactivation seems quite robust. Since BI consists of an extra language conversion stage that none of the other three tasks involve, it is very likely that the right frontotemporal gamma synchronization mirrors the underlying mechanism for BI, or at least part of such mechanism.

Although our experiment design is novel, we are not among the first to discover TGC synchronization during a cognitive task. TGC has already been found in animals for over two decades (cf. Jensen and Colgin, 2007). In humans TGC was later observed and linked to visual short-term memory (Sauseng et al., 2009), spatial memory (Park et al., 2011), working memory (Park et al., 2013), the binding of visual perceptual features (Köster et al., 2018), and verbal long-term memory formation (Lara et al., 2018). Our findings corroborated the mnemonic function of frontal/temporal TGC reported by previous research in that all the three overt tasks in our experiment involve demanding mnemonic processes, including long-term memory for L1S and working memory for L2SH and BI.

Note that the above mentioned results by others were all based on visual stimuli. Wang et al. (2014) administered a speech perception task in EEG and analyzed the TGC. They presented the participants with pictures and auditory stimuli and asked them to judge whether the word matches the picture. Greater TGC was observed in the frontal and left-temporal areas in the match condition only, which the authors took as suggesting an integration of bottom-up and top-down information processing during speech perception. This result is more relevant to our study as it was based on an auditory paradigm. Other research that reported results similar to ours was done by Köster et al. (2014). In that study, researchers implemented a picture encoding and retrieval experiment, and found that prefrontal theta and parietal gamma was related to the controlled retrieval of sequential information of a former event, or episodic retrieval, which is very similar to the TGC pattern for L1S in the current study.

On the other hand, however, the predicted alpha/beta turned out very weak in the raw TF dynamics and was even missing in the subtraction plots. As noted, there is some consensus on the role of alpha/beta as the storage of syntactic phrases in verbal working memory (cf. Meyer, 2018). The question is, therefore, how to explain the alpha/beta absence in these results. One possibility is that in L1S and BI the participants already had sufficient linguistic units before opening their mouth to speak (both tasks preceded by a preparation period), thus no or very little workload for structuring and restructuring syntactic units while they were performing the real oral task, coupled by the fact that both conditions were in L1, which was highly automatic process for the brain. For L2SH, although participants were speaking in L2 and there was some working memory load, the auditory input was syntactically correct and well-organized. As such, they did not have to pin the material to their working memory as we told them not to remember but to keep pace with the audio. This was also manifested on the TF subtraction maps (Figure 3), where L2SH had higher alpha power than L1S and BI, albeit not statistically significant. Another explanation is related to our experiment design. We asked our participants to close their eyes during the experiment in order to exclude visual confounds and reduce artifacts, but some studies have discovered that alpha power varies hugely in eyes-closed and eyes-open experiments (Barry et al., 2007; Putilov and Donskaya, 2014). Which possibility is more plausible to explain our findings still needs further research.

## Limitations and Future Research

Several limitations of the current research warrant consideration, the first of which is the overt experiment mode. Although the overt speaking tasks had never been adopted in EEG studies of interpreting, and therefore can be revealing, they inevitably produced motor noise for the EEG data in the present research. Our preprocessed data could still contain a tiny amount of artifacts, even if the most strict data cleaning measures were applied. Secondly, the method of condition-wise subtraction may have overlooked other mechanisms involved in bilingual tasks. For example, though we asked participants to only comprehend the audio in the L2L task and not to give any response, there still might be a silent interpreting process involved in such task. Therefore BI minus L2L may reflect either the overt interpreting mechanism, or merely the motor process of language production. Besides these, our sample size was quite limited and we did not measure participants’ cognitive abilities such as working memory capacity. There’s also the limitation of language-pair selected in this research.

Future studies can compare overt speaking with silent design to further validate the results of the current study. The current paradigm should also be replicated on other language pairs, for instance, the German-English language pair. Future research should also explore the effects of local features of the stimuli (such as the acoustic traits of the audio) as well as the language backgrounds of the participants (how many languages they speak) on oscillations, which were not analyzed in the current study.

## Conclusion

This is the first study showing the TF dynamics between BI and other sentence-level auditory-oral language activities. We found significantly higher gamma band and delta-theta band TF power values in overt motor language tasks, particularly in BI, relative to auditory comprehension task. We also found right frontocentral theta and parieto-occipital gamma synchronization for articulating self-generated ideas, as compared to the frontocentral theta and midfrontal gamma network for non-self generated ideas. Most importantly, we found for the first time, distinct TGC patterns in BI, i.e., a robust right frontotemporal gamma coactivation, that may indicate the fundamental neural workings of that unique, difficult language task. Our findings could help inform interpreter training and the treatment of interpreting/translation-related aphasia. For example, equipment can be employed to physically stimulate the right frontotemporal region at 40 Hz to strengthen the neuronal network of interpreting.

## Data Availability Statement

The raw data supporting the conclusions of this article will be made available by the authors, without undue reservation.

## Ethics Statement

The studies involving human participants were reviewed and approved by The University of Auckland Human Participants Ethics Committee (Ref. 022991). The patients/participants provided their written informed consent to participate in this study.

## Author Contributions

YZ designed the protocol, implemented the experiment, collected and analyzed the data, and drafted the manuscript. IK helped with the analysis methods and revised the manuscript. TC helped with coding and plotting. MO’H revised the manuscript. KW coordinated the whole project and revised the manuscript. All authors contributed to the article and approved the submitted version.

## Conflict of Interest

The authors declare that the research was conducted in the absence of any commercial or financial relationships that could be construed as a potential conflict of interest.

## Publisher’s Note

All claims expressed in this article are solely those of the authors and do not necessarily represent those of their affiliated organizations, or those of the publisher, the editors and the reviewers. Any product that may be evaluated in this article, or claim that may be made by its manufacturer, is not guaranteed or endorsed by the publisher.

## Footnotes

1
https://www.oxfordenglishtesting.com/

## Appendix

Sentence stimuli used for the BI task:

S1: The United Kingdom research councils are establishing their first overseas office in Beijing.
S2: Over the next several months, let us do what Americans have always done, and build a better world for our children and our grandchildren.
S3: America’s economy is the fastest growing of any major industrialized nation.
S4: To make our economy stronger and more dynamic, we must prepare a rising generation to fill the jobs of the twenty first century.
S5: And I do not mistrust the future, I do not fear what is ahead, for our problems are large, but our heart is larger, our challenges are great, but our will is greater.
S6: To make our economy stronger and more productive, we must make healthcare more affordable.
S7: Baseball is the most popular sport in the United States. It is played throughout the spring and summer, and professional baseball teams play well into the fall.
S8: The year was 1982. The IBM personal computer had only been born the year before.
S9: In 1942, only one out of every 25 high schools in America offers science courses.
S10: With a healthy growing economy, with more Americans going back to work, with our nation and active force for good in the world, the state of our union is confident and strong.
